# Evading Doxorubicin-Induced Systemic Immunosuppression Using Ultrasound-Responsive Liposomes Combined with Focused Ultrasound

**DOI:** 10.3390/pharmaceutics14122603

**Published:** 2022-11-25

**Authors:** Jeongjin Lee, Wooram Um, Hyungwon Moon, Hyeyeon Joo, Yeari Song, Minsung Park, Been Yoon, Hyun-Ryoung Kim, Jae Hyung Park

**Affiliations:** 1 Department of Health Sciences and Technology, SAIHST, Sungkyunkwan University, 81 Irwon-ro, Seoul 06351, Republic of Korea; 2 Department of Biotechnology, Pukyong National University, 45 Yongso-ro, Busan 48513, Republic of Korea; 3 R&D Center, IMGT Co., Ltd., 172 Dolma-ro, Seongnam 13605, Republic of Korea; 4 School of Chemical Engineering, Sungkyunkwan University, 2066 Seobu-ro, Suwon 16419, Republic of Korea

**Keywords:** doxorubicin, immunogenic cell death, cancer immunotherapy, immune checkpoint blockade

## Abstract

Doxorubicin (DOX) is a representative anticancer drug with a unique ability to induce immunogenic cell death of cancer cells. However, undesired toxicity on immune cells has remained a significant challenge, hindering the usage of DOX in cancer immunotherapy. Here, we report a combined therapy to avoid the off-target toxicity of DOX by adapting ultrasound-responsive liposomal doxorubicin and focused ultrasound exposure. Histological analysis demonstrated that the combined therapy induced less hemosiderosis of splenocytes and improved tumor infiltration of cytotoxic T lymphocytes. Additionally, in vivo therapeutic evaluation results indicate that the combined therapy achieved higher efficacy when combined with PD-1 immune-checkpoint blockade therapy by improving immunogenicity.

## 1. Introduction

Doxorubicin (DOX), an antibiotic in the anthracycline group, is one of the most broadly used anticancer drugs. Traditionally, its mode of action is preferential induction of phagocytic clearance, mediated by inhibiting topoisomerase, leading to DNA destabilization [1]. Accordingly, DOX-induced cell death has been considered tolerogenic, occurring with no further immune response [2]. Meanwhile, recent evidence has suggested that DOX may elicit antitumor immunity in certain cancers by causing immunogenic cell death (ICD) through induction of endoplasmic reticulum stress [3,4]. ICD is a differentiated type of cell death that provokes an antigen-specific immune response by releasing damage-associated molecular patterns from dying cells, offering an opportunity to recruit and mature antigen presenting cells (APCs) to allow antigen presentation and a subsequent cytotoxic T lymphocyte (CTL) response [5,6]. Moreover, DOX may contribute to perturbation of the immunosuppressive microenvironment of cancers by eliminating myeloid-derived suppressor cells that inhibit T cells and aid tumor promotion [7,8]. Therefore, DOX can provide multiple antitumor effects not only through direct tumoricidal effects, but also through the immune response. Thus, DOX-induced ICD has emerged as a promising candidate for cancer immunotherapy in recent years [9]. However, suppression of immune cells caused by DOX off-target toxicity has remained a challenge to harnessing the potential of DOX for prompting a systemic immune response against cancer [10,11,12]. In recent decade, several strategies have been reported to suggest targeted delivery of DOX to the tumor by responding to ultrasound. For example, combining focused ultrasound (US) and ThermoDox^®^, a temperature-sensitive liposomal DOX, provides US-triggered release of DOX in the tumor site by local thermal elevation allowing disruption of liposomes [13,14,15].

**Figure 1:**
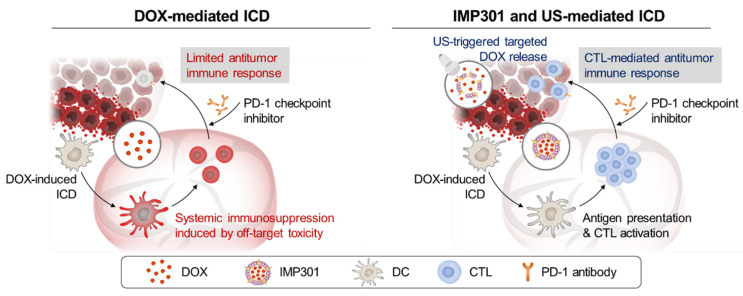
Schematic illustration of DOX-mediated ICD and IMP301 and US-mediated ICD.

Here, we report a novel approach to evade the systemic immunosuppression caused by DOX-induced immune cell toxicity. We assumed that the targeted delivery of DOX using US-responsive liposomes and tumor-specified, focused US exposure could improve the immunogenicity of DOX for antitumor immune response. This would reduce the off-target toxicity on immune cells and, thereby, immunosuppression (Figure 1). To achieve targeted DOX delivery, we used US-responsive liposome IMP301, whose physicochemical properties and sono-responsiveness were extensively investigated in a previous study [16,17]. We set up a 1.5 MHz US instrument with US-triggered DOX release. We examined the therapeutic efficacy of combined treatment with IMP301 and US compared to DOX treatment in the 4T1 murine cancer model and explored the effects of IMP301-mediated targeted DOX delivery on the antitumor immune response. Additionally, we investigated the effects of this combined treatment in PD-1 immune-checkpoint blockade therapy.

## 2. Materials and Methods

### 2.1. Materials

1,2-Distearoyl-sn-glycero-3-phosphocholine (DSPC), (N-(carbonyl-methoxypolyethylene glycol-2000)-1,2-distearoyl-sn-glycero-3-phosphoethanolamine, and sodium salt) (DSPE-mPEG2000), and 1,2-dioleoyl-sn-glycero-3-phosphoethanolamine (DOPE) were purchased from Lipoid AG (Steinhausen, Switzerland). 1-Stearoyl-2-lyso-sn-glycero-3-phosphocholine (MSPC, S-LysoPC) was purchased from NOF America Corporation (White Plains, NY, USA). Doxorubicin hydrochloride (DOX) was purchased from Gemini Pharmaceuticals Inc. (Hauppauge, NY, USA). Cholesterol, ammonium sulfate, L-histidine, paraformaldehyde, and bovine serum albumin (BSA) were purchased from Sigma-Aldrich (St. Louis, MO, USA). Sucrose was purchased from CheilJedang (Seoul, Korea). Murine mammary carcinoma (4T1) cell lines were obtained from the American Type Culture Collection (ATCC, Rockville, MD, USA). For cell growth, we purchased RPMI 1640 and fetal bovine serum (FBS) from Capricorn Scientific (Ebsdorfergrund, Germany). The antibiotic-antimycotic solution, 0.25% trypsin-EDTA, and Dulbecco’s phosphate-buffered saline (DPBS) were purchased from Welgene (Daegu, Korea). Anti-PD1 antibody was obtained from BioXcell (Lebanon, NH, USA). FITC anti-mouse CD80 antibody was purchased from Biolegend (San Diego, CA, USA). eFluor^TM^660 anti-CD8a antibody was obtained from Invitrogen (San Diego, CA, USA). All the materials were used without further purification.

### 2.2. Preparation of IMP301

IMP301 was synthesized using a previously reported procedure [{Kim, 2022 #183}]. In brief, the lipid solution was prepared by dissolving 1.5 g of DSPC, 2.66 g of DSPE-PEG, 2.20 g of cholesterol, 9.16 g of DOPE, and 0.50 g of MSPC in 62.5 mL of ethanol. The lipid solution was then heated to 60 °C and dropped into 437.5 mL of stirred 250 mM ammonium sulfate solution. The lipid solution was serially extruded at 50 °C and dialyzed with 10% sucrose (pH 6.5) and a 10 mM histidine buffer using a dialysis membrane with a 12–14 kDa cut-off. Additionally, DOX was loaded into liposomes using the ammonium gradient method at a 1:8 volume ratio. The resulting solution was stirred at 37 °C for 2 h, and then the DOX concentration was adjusted to 2 mg/mL.

### 2.3. In Vitro Release Behavior of IMP301

Free DOX and IMP301 (DOX 2 mg/mL, 1 mL, *n* = 4) were prepared in a dialysis membrane bag (molecular weight cut off = 12–14 kDa) to evaluate DOX release from IMP301 by US irradiation. Next, the membrane bag was immersed in PBS (pH 7.4) and moved to a 3% agarose mold. The pre-produced agarose mold consisted of TPX film (Yusang, Korea) with an inner diameter of 15 mm and an outer diameter of 30 mm. Each membrane bag was exposed to US using a wet-type, high-intensity-focused US system (VIFU-2000, Alpinion Medical System, Seoul, Korea). The US exposure time was 120 s (power, 80 W; duty cycle, 2%; pulse repetition frequency, 250 Hz). After US irradiation, each sample was gently shaken at 37 °C and 120 rpm. According to predetermined time intervals, the membrane bag was transferred to fresh medium, and the DOX concentration was measured using a UV-vis spectrometer (Agilent 8453 UV-visible spectroscopy system, Agilent Technology, Santa Clara, CA, USA) at 480 nm.

### 2.4. Animal Models

All animal experiments were conducted at Sungkyunkwan University and were revised and approved by the Institutional Animal Care and Use Committees of Sungkyunkwan University (SKKUIACUC2021-09-64-1). All in vivo experiments were performed using metastasis-mimicking biolateral 4T1 tumor-bearing mice, and 4T1 cells were cultured in RPMI 1640 media containing 10% FBS and antibiotic-antimycotics (100 U/mL) in a humidified CO_2_ incubator at 37 °C. The animal model was established by injecting 1 × 10^6^ 4T1 cells into the left flank of BALB/c mice (female, five weeks old) on day 0. An in vivo anti-tumor treatment was applied when the tumor volume reached 55–60 mm^3^. Additionally, a secondary tumor site was inoculated by injecting 10^6^ 4T1 cells into the right flank to mimic metastatic conditions. Tumor volume was calculated as the largest diameter × smallest diameter^2^ × 0.5.

### 2.5. In Vivo Antitumor Therapy

To observe the therapeutic efficacy of US-responsive anti-tumor drug release, we divided the mice into three groups on day 7: Control, DOX, and IMP301+US groups (*n* = 5 in each group). DOX or IMP301 (3 mg kg^−1^ of DOX) was administered intraveneously on days 7, 10, and 13. The treatment area was specified by using US imaging system ECUBE9 (B-mode, 4 MHz, S12-4 transducer, Alpinion Medical System, Korea). A central focal point was set in the center of the tumor, and then six more points were set in the vicinity with 2 mm of X, Y intervals to apply US to a total of seven focal points. US was irradiated to the tumor site 1 h after injection under the following conditions: power, 80 W; duty cycle, 2%; pulse repetition frequency, 250 Hz; 7 focal points; 20 s per point). The tumor volume and body weight were recorded every two days.

### 2.6. Immunohistochemistry

Tumor tissues were collected on day 15 and fixed with 4% paraformaldehyde. The tissues were embedded in paraffin and sectioned into five-μm-thick sections on glass slides. The sections were deparaffinized and stained with fluorescent conjugated antibodies (FITC conjugated anti-mouse CD80 antibody and eFluor660™ conjugated anti-mouse CD8 antibody). The antibodies were diluted in blocking solution (1:200, 1% BSA in PBS) and incubated overnight at 4 °C before DAPI staining. The stained slides were imaged with a confocal laser scanning microscope (Leica TCS SP8; Leica Microsystems, Wetzlar, Germany).

### 2.7. Ex Vivo Histology

The spleen, liver, lungs, and kidneys of the mice were collected on day 30 and fixed with 4% paraformaldehyde solution. The tissues were embedded in paraffin and divided into five-μm-thick sections on glass slides. A microscope slide scanner (Axio Scan. Z1, Carl Zeiss, Germany) was used to observe the hematoxylin and eosin-stained tissue slides.

### 2.8. In Vivo Anti-Tumor Efficacy Test with Immune Checkpoint Blockade

To evaluate the synergistic effect of the combination therapy with anti-PD1 antibody (aPD1), we divided the mice into three groups: aPD1, DOX+aPD1, and IMP301+US+aPD1 groups (*n* = 4 in each group) on day 7. DOX or IMP301 (3 mg kg^−1^ DOX) was administered intraveneously on days 7, 10, and 13. 1 h After 1 h from injection, the tumor was exposed to US (power, 80 W; duty cycle, 2%; pulse repetition frequency, 250 Hz; and Time, 140 s). Anti-PD1 antibody (3 mg kg^−1^ per mouse) was injected intraperitoneally after one additional day. The tumor volume and body weight were recorded every two days.

### 2.9. Statistics

All the data in this article were represented as the mean ± standard deviation (or standard error). Statistical analysis was performed using GraphPad Prism (GraphPad Software, San Diego, CA, USA). The data were analyzed using one-way ANOVA. The detailed pre-processing of data and sample size for each analysis are shown in the caption.

## 3. Results and Discussion

### 3.1. In Vivo Immunogenicity and Systemic Toxicity of the Combination of IMP301 and US Treatment

IMP301 is US-sensitive liposomal DOX developed for improving DOX delivery efficiency into the tumor and reducing off-target toxicity in cancer therapy by securing DOX in the absence of US [16]. IMP301 has a 94.23 ± 24.51 nm of Z-average and 12.22 ± 0.33% of DOX loading content (Figures S1 and S2). The amount of released DOX was quantitatively assessed in the presence or absence of US to verify the US responsiveness of IMP301 (Figure S3). In the absence of US, IMP301 released 26.5 ± 4.28% of its DOX content at 48 h. In contrast, the amount of released DOX was significantly higher at 55.3 ± 3.54 in the presence of US. Meanwhile, DOX was 99% released within 48 h, and there were no significant differences in the release kinetics after irradiating with US. Given the US responsiveness of IMP301, we assumed that the combination of IMP301 and US treatment (IMP301+US) could reduce the off-target release of DOX by securing DOX in the absence of US.

**Figure 2:**
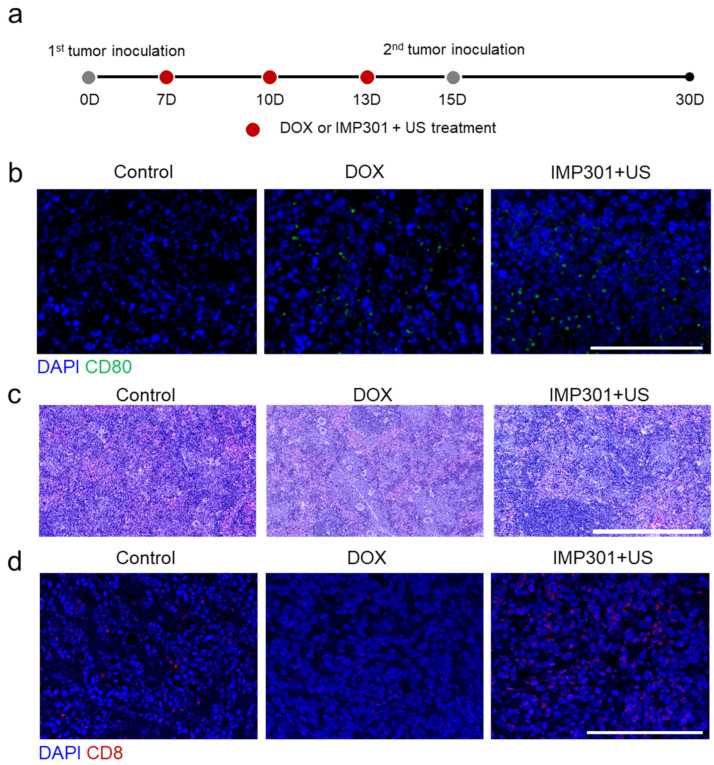
In vivo immunogenicity and systemic toxicity of the combination of IMP301 and US treatment. (**a**) Schematic illustration of the treatment protocol and (**b**) immunohistochemistry of mature APCs in tumor tissues. Blue: DAPI, Green: CD80. Scale bar represents 100 µm. (**c**) Hematoxylin and eosin-stained spleen tissues. Scale bar represents 500 µm. (**d**) Immunohistochemistry of infiltrated CTLs in tumor tissues. Blue: DAPI, Green: CD8. Scale bar represents 100 µm.

After establishing the potential of IMP301+US in tumor-targeted delivery of DOX, we evaluated APC maturation, splenocyte damage, and CTL infiltration using 4T1 tumor-bearing mice to assess the immunogenicity of IMP301+US according to the treatment protocol illustrated in Figure 2a. To maximize the release of DOX into the tumor, we tried to examine the ultrasound when IMP301 was the most accumulated on the tumor. According to the preliminary study, it was observed that IMP301 reached the tumor the most at 1 h after being intravenously injected into the body (Figure S4a). Furthermore, applying the US 1 h after injection led to the improved accumulation of DOX into the tumor (Figure S4b). Therefore, we applied US into the tumor after 1 h from IMP301 injection.

Briefly, IMP301+US was applied to 4T1 tumor-bearing mice three times according to a three-day interval from when the tumor volume reached 55–60 mm^3^. As shown in Figure 2b, CD80 fluorescence signals (green) were observed in DOX-treated tumors and IMP301+US-treated tumors, suggesting that IMP301+US successfully provoked APC maturation by causing DOX-mediated ICD. Meanwhile, DOX stimulates APCs and damages lymphocytes via hemosiderosis by interfering with intracellular iron homeostasis [1,18]. Hemosiderosis is an overload of iron in cells and is frequently considered a cause of CTL generation impairment [19,20]. As expected, Figure 2c shows that less hemosiderosis was observed in the spleen of the IMP301+US-treated mice than that of DOX-treated mice exhibiting remarkable hemosiderosis. The results indicate that IMP301+US can reduce the off-targeted delivery of DOX into the spleen and, thereby, induce less hemosiderosis than DOX treatment. In addition, a higher CD8 fluorescence signal (red) from the IMP301+US-treated tumor than from the DOX-treated tumor suggests that reduced lymphocyte hemosiderosis allowed increased tumor infiltration of CTLs (Figure 2d). Overall, these results suggest that IMP301+US has the potential to elicit anti-tumor immunity by inducing ICD. Furthermore, preventing the off-target release of DOX allows improved tumor infiltration of CTLs by reducing splenocyte damage.

### 3.2. In Vivo Therapeutic Efficacy of the Combination of IMP301 and US in 4T1 Tumor-Bearing Mice

**Figure 3:**
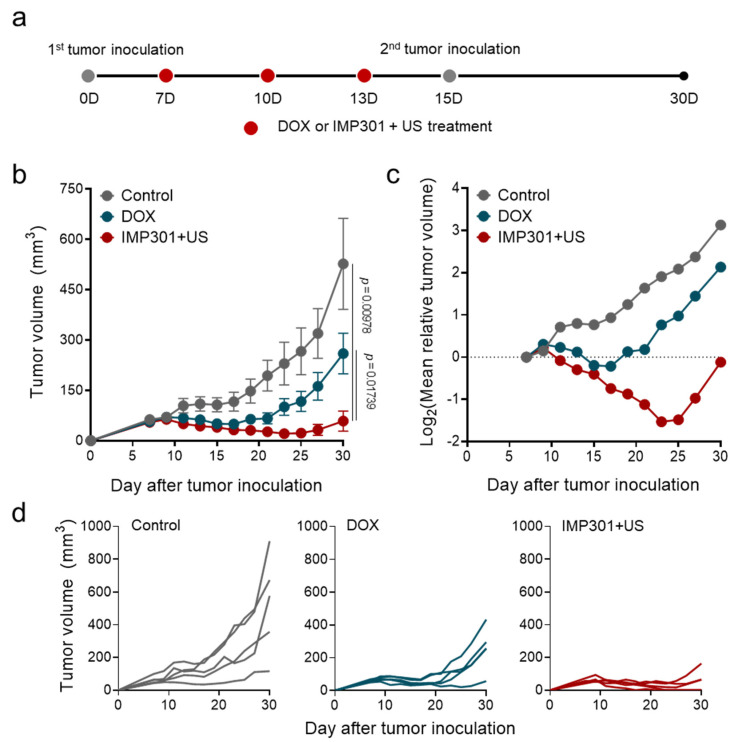
In vivo therapeutic efficacy of the combination of IMP301 and US in 4T1 tumor-bearing mice. (**a**) Schematic illustration of the treatment protocol; (**b**,**c**) changes in tumor size as a function of time. (**d**) Individual tumor growth in volume (*n* = 5).

**Figure 4:**
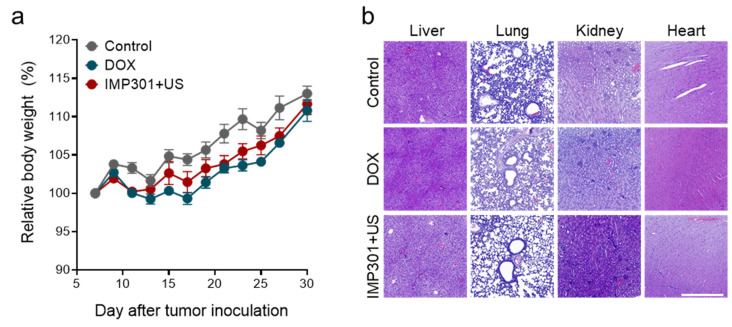
In vivo toxicity of the combination of IMP301+US. (**a**) Changes in body weight and (**b**) H&E-stained liver, lung, kidney, and heart tissues. Scale bar represents 500 µm.

On the basis of the potential to provoke anti-tumor immunity, we evaluated the therapeutic efficacy of IMP301+US in 4T1 tumor-bearing mice according to the treatment protocol illustrated in Figure 3a. In a previous study using flank tumor-bearing mice, it was confirmed that IMP301 showed slight DOX release in the absence of US at the tumor site, while time-dependently increased DOX accumulation at the tumor after US irradiation. In addition, due to the selective release of DOX to the tumor due to US-responsiveness, it was confirmed that IMP301+US showed excellent tumor growth inhibitory efficacy compared to the IMP301 administration group. In this study, we comparatively evaluated the therapeutic efficacy of DOX and IMP301+US-treated groups [16]. After 30 days from tumor inoculation, DOX suppressed 50.75% of tumor growth (526.99 ± 303.55 mm^3^ to 259.50 ± 134.73 mm^3^) and delayed tumor doubling time from 10.41 days to 15.10 days compared to the control (Figure 3b–d). Notably, IMP301+US showed significantly improved therapeutic efficacy relative to DOX treatment. In comparison with the control, IMP301+US suppressed 88.89% (58.52 ± 66.86 mm^3^) of tumor growth and delayed tumor doubling time to more than 20 days, which could be attributed to reduced hemosiderosis and improved CTL infiltration into the tumor. Meanwhile, no significant weight reduction or distinctive pathological signs in the liver, lung, or kidney were observed, as shown in Figure 4a,b.

### 3.3. In Vivo Therapeutic Efficacy of the Combination of aPD1, IMP301, and US Treatment in 4T1 Tumor-Bearing Mice

**Figure 5:**
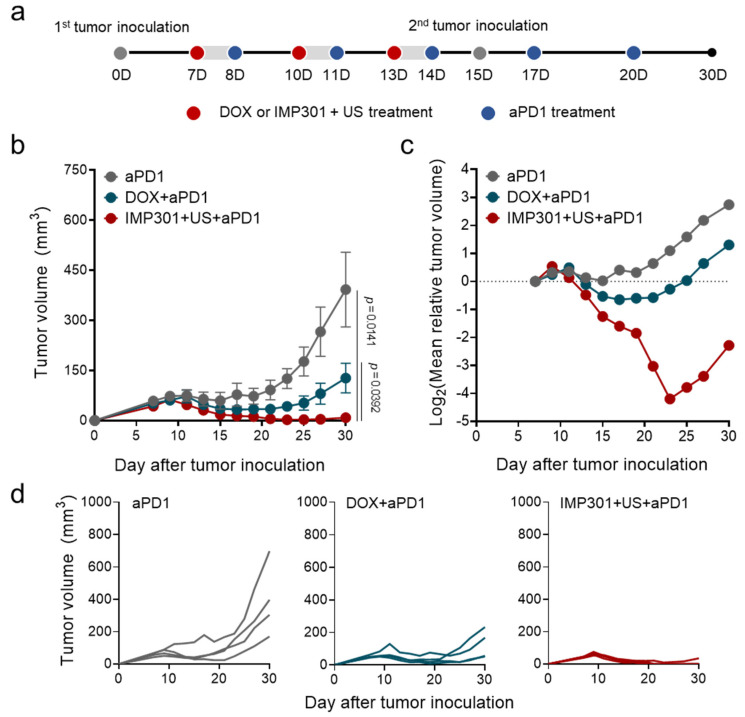
In vivo therapeutic efficacy of the combination of aPD1, IMP301, and US treatment in 4T1 tumor-bearing mice. (**a**) Schematic illustration of the treatment protocol; (**b**,**c**) changes in tumor size as a function of time. (**d**) Individual tumor growth in volume (*n* = 4).

Finally, we evaluated the therapeutic potential of IMP301+US by co-administrating PD-1 checkpoint blockade according to the treatment protocol as illustrated in Figure 5a. Given the potential of IMP301+US to improve tumor infiltration of CTLs, we selected PD-1 checkpoint blockade as an adjuvant therapy to augment the immunogenic potential of IMP301+US by normalizing CTL-mediated cancer rejection. In addition, we established a bilateral 4T1 tumor model by injecting a secondary tumor into the opposite side of the primary tumor on day 15 to mimic metastatic conditions. After 30 days from tumor inoculation, DOX suppressed 57.2% of tumor growth (392.30 ± 223.32 mm^3^ to 127.32 ± 88.45 mm^3^) and delayed tumor doubling time from 15.56 days to 21.08 days compared to the PD-1 checkpoint blockade-treated group (aPD1) (Figure 5b,c). Notably, IMP301+US suppressed 97.7% of tumor growth (8.84 ± 17.68 mm^3^)and delayed tumor doubling time by more than 20 days compared with aPD1 (Figure 5b–d). Furthermore, IMP301+US eliminated established tumors in 75% of mice (3/4), which might be attributed to improved immunogenicity relative to DOX (0/4).

## 4. Conclusions

In this study, we evaluated the therapeutic potential of IMP301 in combination with US in the context of ICD-mediated antitumor immune response. Compared to DOX, IMP301+US more efficiently promoted APC maturation and DOX-mediated ICD and CTL infiltration into the tumor by reducing splenic damage. Overall, these results indicate that IMP301+US could evade doxorubicin-induced systemic immunosuppression by US-triggered targeted DOX delivery into the tumor. As a result, IMP301+US can effectively inhibit tumor growth compared to DOX with or without PD1 immune checkpoint treatment by reducing hemosiderosis of splenocytes and improving CTL tumor infiltration. Recently, sono-immunotherapy, an ultrasound-mediated cancer immunotherapy, is emerging as a promising candidate due to the precision, safety, and high accessibility of US in the clinic [21,22,23,24]. It is noteworthy that US can improve the therapeutic potential of drugs by facilitating endocytosis or perturbing cellular functions via acoustic cavitation [25,26,27]. Meanwhile, recent studies on liposomes suggest potential ways to improve the therapeutic efficacy of IMP301+US. For example, combining US-sensitive liposomes and focused US has the potential in treating brain tumors by disrupting the brain-blood barrier [28]. Meanwhile, adding peptidal PD-L1 to liposome can allow the rejuvenation of cytotoxic T cell-mediated antitumor immunity [29]. On the other hand, microbubble-based carriers can provide a theranostic modality including targeted DOX delivery and contrast-enhanced imaging [30,31]. Therefore, IMP301+US may offer a promising therapeutic option for ICD-mediated cancer therapy or concomitant therapy with another cancer immunotherapy, such as immune checkpoint blockade therapy.

## Data Availability

Not applicable.

## Supplementary Materials

The following supporting information can be downloaded at: https://www.mdpi.com/article/10.3390/pharmaceutics14122603/s1, Figure S1: Physicochemical characterization of IMP301 (*n* = 3); Figure S2: Size distribution of IMP301 (*n* = 3); Figure S3: In vitro release profiles of DOX and IMP301 (*n* = 4); Figure S4: In vivo biodistribution of IMP301; Figure S5: In vivo therapeutic efficacy of the US in 4T1 tumor bearing mice.
