# Supplementary material for: Evading Doxorubicin-Induced Systemic Immunosuppression Using Ultrasound-Responsive Liposomes Combined with Focused Ultrasound
Source: Pharmaceutics. 2022 Nov 25;14(12):2603. doi: 10.3390/pharmaceutics14122603 (PMC9784431; doi:10.3390/pharmaceutics14122603)
Supplement: Supplementary file 1 [file pharmaceutics-14-02603-s001.zip › pharmaceutics-1939219-supplementary.pdf]

**Supplementary Materials**

| Size distribution | | | Loading efficacy (%) | Loading contents (%) |
|---|---|---|---|---|
| Z-average (d. nm) | PDI | STD (d. nm) | | |
| 94.23 | 0.052 | 24.51 | 97.8 ± 2.6 % | 12.22 ± 0.33 % |

**Figure S1.** Physicochemical characterization of IMP301 (*n* = 3).

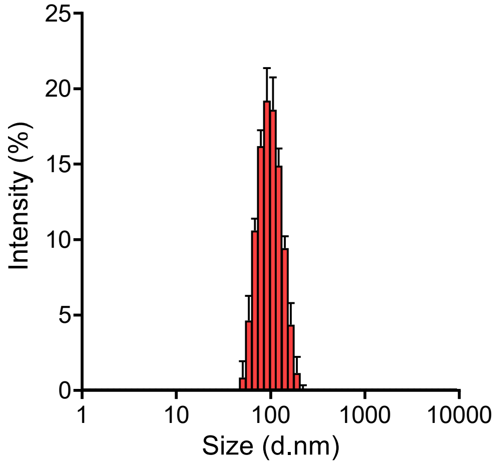


**Figure S2.** Size distribution of IMP301 (*n* = 3).

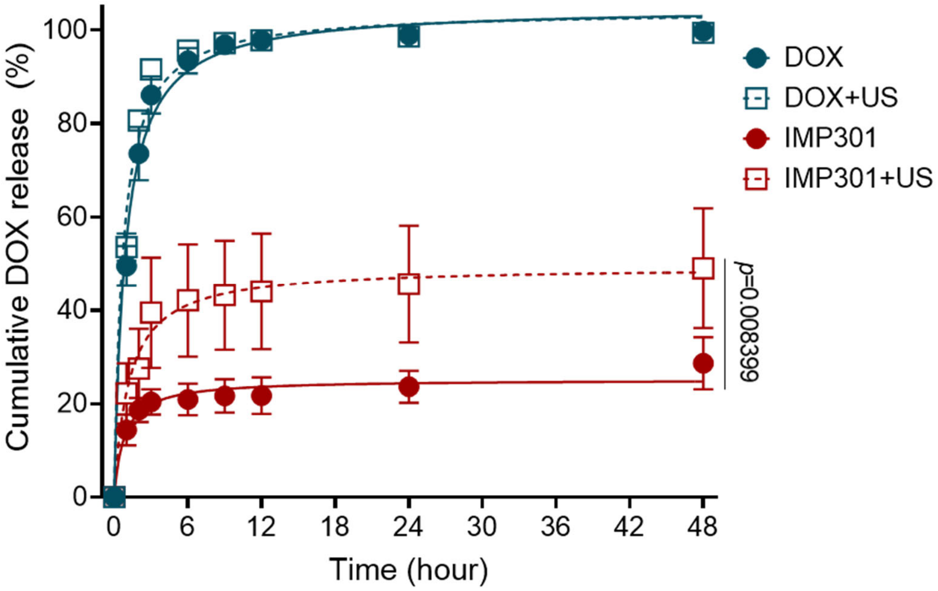


**Figure S3.** *In vitro* release profiles of DOX and IMP301 (*n* = 4).

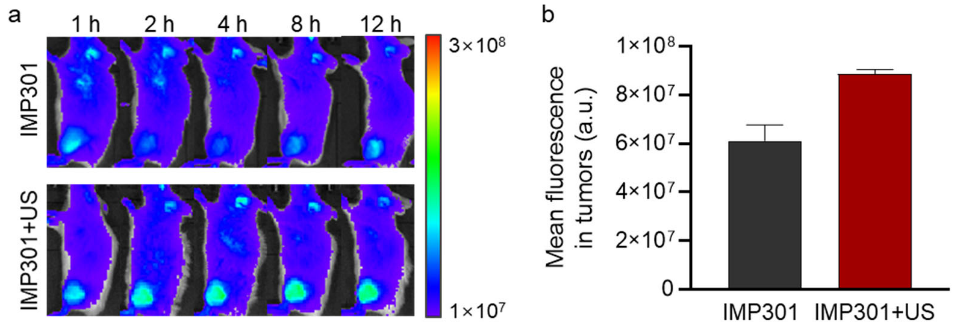


**Figure S4. *In vivo* biodistribution of IMP301**. (a) *In vivo* fluorescence images after injecting IMP301 with or without US irradiation. (b) Quantification of the fluorescence intensity of DiD in the tumor at 4 h ($n = 3$).

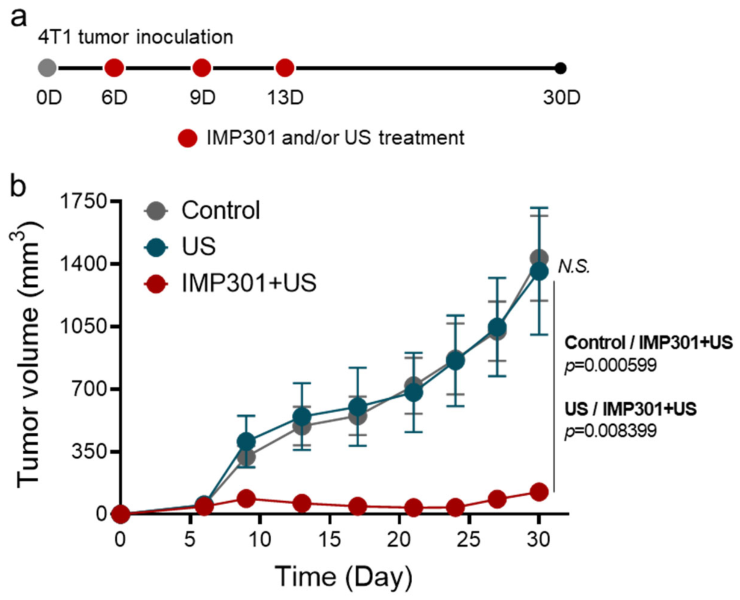


**Figure S5. In vivo therapeutic efficacy of the US in 4T1 tumor bearing mice.** (a) Schematic illustration of the treatment protocol. (b) Changes in tumor size as a function of time ($n = 5$).
